# Emerging and Re-Emerging Infectious Diseases: Humankind’s Companions and Competitors

**DOI:** 10.3390/microorganisms10010098

**Published:** 2022-01-04

**Authors:** Nikolaos Spernovasilis, Sotirios Tsiodras, Garyphallia Poulakou

**Affiliations:** 1 School of Medicine, University of Crete, 71003 Heraklion, Greece; 2 Fourth Department of Internal Medicine, Attikon University Hospital, 12462 Athens, Greece; sotirios.tsiodras@gmail.com; 3 School of Medicine, National and Kapodistrian University of Athens, 11527 Athens, Greece; gpoulakou@gmail.com; 4 Third Department of Internal Medicine, Sotiria General Hospital, 11527 Athens, Greece

Infectious disease outbreaks had a significant impact on shaping the societies and cultures throughout human history. Major epidemics and pandemics such as the bubonic plague, yellow fever, cholera, typhus, and influenza have afflicted humanity over the centuries by determining outcomes of wars, extinguishing empires, and wiping out entire populations [1]. In the 1960s and 1970s, when the public health’s attention had shifted away from acute infections to chronic illnesses because of the emergence of antibiotics and vaccines, the belief that infectious diseases had been defeated was widespread [2,3]. The subsequent epidemics and pandemics, especially the one due to the HIV-1 virus, deconstructed that belief and reminded us of the threats that infectious diseases continued to pose to human health. However, it seems that lessons from these outbreaks were not well learned, and history repeated itself in the case of SARS-CoV-2, resulting in the severe global impact of the COVID-19 pandemic.

Emerging infectious diseases (EIDs) and re-emerging infectious diseases (REIDs) are responsible for a significant proportion of the infectious disease outbreaks that have plagued humanity over the ages. EIDs are infectious diseases that have not occurred in humans before, have occurred previously in humans but affected only small populations in isolated areas, or have occurred in the past but were only recently recognized as distinct diseases caused by infectious agents [4]. REIDs are infectious diseases that constituted significant health problems in a particular geographic area or globally during a previous time period, then declined greatly, but are now again becoming health problems of major importance [4]. Remarkably, specific infectious pathogens, such as influenza viruses, continuously emerge and re-emerge.

Most EIDs and REIDs have a zoonotic origin, denoting that the disease has emerged from an animal and crossed the species barrier to infect humans [5]. The majority of these zoonoses come from wildlife, while others originate from domesticated animals and intensive animal farming [6,7,8]. They are transmitted from animals to humans through direct contact, droplets, water, food, vectors, or fomites [9]. However, as mentioned, not all EIDs and REIDs are zoonoses. Infections due to several multi-drug resistant organisms, such as vancomycin-resistant *Staphylococcus aureus* and *Candida auris*, are considered non-zoonotic EIDs that are related to antibiotic overuse and misuse. Irrespective of the origin of an EID or REID, in order for a pathogen to be established in the community, it must be introduced into a vulnerable population and have the ability to spread from human to human and cause disease [10].

The substantial developments in surveillance and diagnostics that have been achieved nowadays led to the realization that the incidence of EIDs and REIDs has increased considerably over the last few decades [11]. These developments improve the detection of outbreaks in general and facilitate the early implementation of response measures. Even after controlling for the reporting effect, the number of EID events worldwide still shows a highly significant relationship with time [7]. Certain geographic areas such as Asia, tropical Africa, and Latin America are more likely to experience EID and REID events [7,12,13,14]. Furthermore, pathogens with a previously distinct geographic distribution can be introduced to new regions more easily these days [15]. This appears to be the case with vector-borne pathogens such as dengue and West Nile viruses, with their expansion to higher latitudes due to climate change and changes in vector distribution [16]. Notably, the aforementioned EID and REID events have diverse potential to give rise to epidemics and pandemics, and their association with critical illness, adverse health outcomes, and the need for isolation measures is variable [17,18,19].

Many factors precipitate the occurrence and transmission of EIDs and REIDs by enabling the infectious agents to evolve in suitable ecological niches, reach and adopt to vulnerable hosts, and spread more easily among their hosts. Such factors include the expanding human population, population aging, urbanization, globalization, climate change, poverty and social inequality, conflicts, migration, wildlife trade and consumption, industrial livestock production, irrational antimicrobial use and the development of resistance in humans and livestock, as well as breaches in implementing public health measures, such as sanitation and vaccination programs (Figure 1; modified from [20]) [20,21,22]. Thus, it is obvious that the emergence and re-emergence of infectious diseases are tightly linked with the scale, pace, intensity, and nature of human activity.

The impact of EIDs and REIDs can be assessed in many different ways, including global mortality and morbidity, economic burden, and social and geopolitical implications. For example, with regard to the COVID-19 pandemic, as of December 2021 there were more than 275 million cases worldwide and more than 5 million recorded deaths [23]. However, the greatest proportion of morbidity (death plus disability) associated with COVID-19 is likely to be due to disability (e.g., long COVID) or delayed deaths due to secondary health sequelae, rather than direct death [24]. Thus, while direct COVID-19 mortality is substantial, it is likely to account for less morbidity (calculated as disability-adjusted life years, DALYs) overall than disability or organ damage in survivors does [24]. It is noteworthy that mental health effects may have huge implications and constitute a pandemic within the pandemic [25]. Regarding the socioeconomic fingerprint of the current pandemic, education, employment, global poverty, public psychology, tourism and the associated industry, global production, logistics, and global trade have all been negatively affected [26]. Although it cannot be quantified precisely, since the pandemic is still ongoing, there is already a huge, unparalleled in human history, loss of economic well-being and social capital, especially among the weaker sections of societies, such as economically and socially deprived citizens.

The above-mentioned discourse showcases the significance of a coordinated and sustained global response to address the threats that EIDs and REIDs pose to humans. This response should be based on a multifaceted approach, under a One Health perspective, which integrates different disciplines and sectors, including veterinary medicine, biology, epidemiology, immunology, human medicine, public health, behavioral and communication science, anthropology, sociology, psychology, education, and others. Focusing on and investing in proactive and preventive strategies and policies, especially in developing countries where resources are limited, along with strengthening surveillance, rapid risk assessment, and risk communication are of paramount importance in order to prevent or detect EIDs and REIDs at an early stage, when more rigorous control options are available. Investing in all aspects of research will lead to a better understanding of the physiology and specific risk factors affecting the spread of high-risk EIDs and REIDs, and to improved preventive and therapeutic interventions.

In this regard, newer epidemiological surveillance tools, such as artificial intelligence [27] and wastewater surveillance [28,29], the evolution of rapid, multiplex, and easy to use diagnostics [30], and the prompt development and evaluation of novel therapeutics with fewer regulatory, legal, and financial hurdles [31], will greatly facilitate a future response. In addition, one of the greatest scientific achievements during the current pandemic was the record-breaking speed in producing safe and effective vaccines against this novel virus, many of which are based on new technology, that have already saved millions of lives across the world [32]. Nevertheless, a critical appraisal of the response to the ongoing COVID-19 pandemic will tremendously assist preparedness efforts for the future. Even if we do not completely succeed in outcompeting our emerging, re-emerging, and continuously emerging “companions”, we may identify ways to harmonize our co-existence in a way that minimizes the loss of human life.

In this Special Issue, entitled “Emerging Infectious Diseases and Strategies for Their Prevention and Control”, the contributions from leading authors in the field are intended to improve our understanding regarding the roots of EIDs and REIDs and to expand our knowledge of the appropriate actions to reduce their negative impact on humanity.

## Figures and Tables

**Figure 1:**
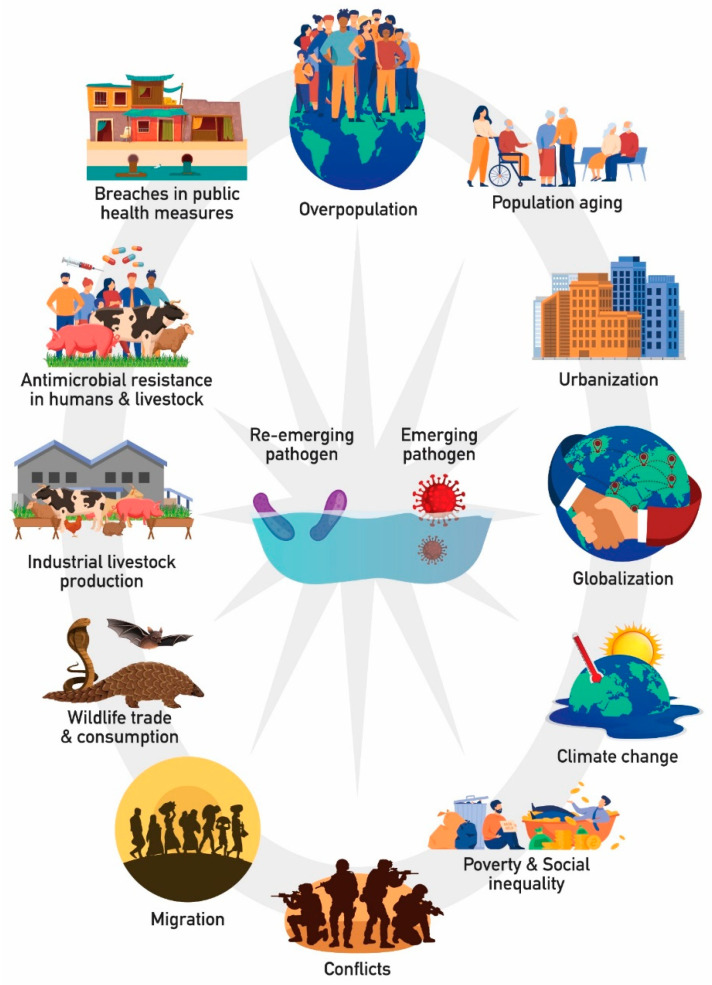
Factors that precipitate the occurrence and transmission of EIDs and REIDs.

## Data Availability

Not applicable.
